# Molecular dynamics simulation of pyruvate kinase to investigate improved thermostability of artificially selected strain in *Enterococcus faecium*

**DOI:** 10.1007/s13258-023-01373-x

**Published:** 2023-04-06

**Authors:** Vladimir Li, Chul Lee, Youngho Lee, Heebal Kim

**Affiliations:** 1 Interdisciplinary Program in Bioinformatics, Seoul National University, Seoul, Republic of Korea; 2 Laboratory of Neurogenetics of Language, The Rockefeller University, New York, NY USA; 3 Department of Agricultural Biotechnology, Research Institute for Agriculture and Life Sciences, Seoul National University, Gwanak-gu 1, Gwanak-ro, Seoul, 08826 Republic of Korea; 4 eGnome, C-1008, H businesspark, 26, Beobwon-ro 9-gil, Songpa-gu, Seoul, Republic of Korea

**Keywords:** *Enterococcus faecium*, In silico, Molecular dynamics, Adaptive laboratory evolution

## Abstract

**Background:**

*Enterococcus faecium* (*E. faecium*) is a member of symbiotic lactic acid bacteria in gastrointestinal tract and it was successfully used to treat diarrhea cases in humans. For a lactobacteria to survive during the pasteurization process, resistance of proteins to denaturation at high temperatures is crucial. Pyruvate kinase (PYK) is one of the proteins possessing such property. It plays a major role during glycolysis by producing pyruvate and adenosine triphosphate (ATP).

**Objective:**

To assess the acquired thermostability of PYK of ALE strain using *in silico* methods.

**Methods:**

First, we predicted and assessed tertiary structures of our proteins using SWISS-MODEL homology modelling server. Second, we then applied molecular dynamics (MD) simulation to simulate and assess multiple properties of molecules. Therefore, we implemented comparative MD to evaluate thermostability of PYK of recently developed high temperature resistant strain of *E*. *faecium* using Adaptive Laboratory Evolution (ALE) method. After 20ns of simulation at different temperatures, we observed that ALE enhanced strain demonstrated slightly better stability at 300, 340 and 350 K compared to that of the wild type (WT) strain.

**Results:**

We collected the results of MD simulation at four temperature points: 300, 340, 350 and 400 K. Our results showed that the protein demonstrated increased stability at 340 and 350 K.

**Conclusion:**

Results of these study suggest that PYK of ALE enhanced strain of *E. faecium* demonstrates overall better stability at elevated temperatures compared to that of WT strain.

## Introduction

PYK is responsible for transferring phosphate group from phosphoenolpyruvate (PEP) to adenosine diphosphate (ADP), thus producing pyruvate and a molecule of adenosine triphosphate (ATP) (Schormann et al. 2019). For lactic acid bacteria, such as *Enterococcus faecium* it is crucial since they mostly rely on glycolysis for energy production in anaerobic conditions (Ramsey et al. 2014). In case PYK malfunctions the bacteria will fail to produce ATP and pyruvate, thus severely limiting its vitality and are likely to cease its functions.

Commercial strains of *E. faecium* are widely used in productions of probiotics (HY07, T110) (Natarajan and Parani 2015; Duan et al. 2019). SF68 strain was proven to effectively treat diarrhea in both children and adults (Buydens and Debeuckelaere 1996). Also, the bacteria are used in fermentation processes of food and production of dairy products, such as cheese (Gelsomino et al. 2002).

However, during food processing procedures, bacteria are subjected to high temperatures (> 60 °C), to which they are not adapted. Thus, development of heat tolerant bacteria is important in preserving their beneficial properties. Adaptive Laboratory Evolution (ALE) is a method to improve bacterial resistance to extreme environment in laboratory environment based on the principles of molecular evolution (Dragosits and Mattanovich 2013). ALE is based on applying principles of natural evolution in the laboratory conditions. During this process, natural selection is shifted towards selected conditions for a population to acquire desired mutations (Dragosits and Mattanovich 2013). Population with deleterious mutations are removed, while those with beneficial mutations are selected for further generations (Loewe and Hill 2010). Multiple ALE enhanced bacterial strains have been developed over a period of time (Dragosits and Mattanovich 2013). Heat (41.5 °C) resistant *E. coli* strain was developed through 2000 generations by Riehle et al. and heat (48.5 °C) resistant *E. coli* strain developed by Rudolph et al. are good examples of how ALE technology can be used in biotechnology to create stress resilient strains (Riehle et al. 2001; Rudolph et al. 2010).

As a preliminary study, Min et al. previously developed ALE an enhanced strain of *E. faecium*, which demonstrated better persistence (up to 75 °C) compared to wild type bacteria (up to 69 °C) (Min et al. 2020). They applied comparative genomic approaches and proposed several proteins, including PYK, as candidates responsible for increased thermostability of the artificially selected strain, but neglected to consider the perspective in structural biology. Although, multiple proteins were proposed, here, we wanted to specifically assess whether PYK gained heat resistance, due to its importance as an enzyme involved in energy production, and thus we performed molecular dynamics (MD) simulation to test whether PYK contributes to the enhanced survivability of the bacteria at elevated temperatures in wild and ALE strains of *E. faecium*.

## Materials and methods

### Protein sequences, 3D modelling and structure assessment

The WT and ALE sequences of PYK were retrieved from the National Center for Biotechnology Information (NCBI, https://www.ncbi.nlm.nih.gov/) under accession numbers QIT61652 and QIT59269 correspondingly. Protein structures were prepared using homology modelling server SWISS-MODEL (Waterhouse et al. 2018). The resulting structures were validated using ProSA-Web server (Wiederstein and Sippl 2007).

### Molecular dynamics simulations

The MD simulations were performed using GROMACS software package (Van Der Spoel et al. 2005; Abraham et al. 2015). We used OPLS (Optimized Potentials for Liquid Simulations) all-atom force-field (Jorgensen and Tirado-Rives 1988; Jorgensen, Maxwell and Tirado-Rives, 1996). The potential functions of this force field are optimized to reproduce experimental data and parameters on fluids, making them computationally efficient and able to adequately describe behavior of proteins in solutions (Jorgensen and Tirado-Rives 1988; Jorgensen, Maxwell and Tirado-Rives, 1996). All systems were brought to electroneutral state through addition of sodium and chloride ions. Simulation was performed with TIP3P water model simulation in rhombic dodecahedron box (Jorgensen et al. 1983). TIP3P (transferrable intermolecular potential 3P) is a 3-site rigid water molecule with corresponding charges and Lennard-Jones parameters assigned to each atom (Jorgensen et al. 1983). Rhombic dodecahedron box was utilized to house the protein and the solvent. It is close to being a sphere, thus making it optimal for studying proteins in solutions, as fewer solvent molecules are required to fill the box (Abraham et al. 2015).

The initial energy minimization (EM) step was followed by equilibration procedure under constant number of particles, volume and temperature (NVT) conditions using by gradually increasing temperature from 300 to 400 K over a course of 500 ps. Hydrogen bonds constraints were applied with LINCS algorithm and V-rescale thermostat was used.

In the next step, constant number of particles, pressure and temperature (NPT) method was applied to further equilibrate the system for 1 ns using Verlet cutoff scheme, V-rescale thermostat and Parrinello-Rahman barostat. Reference pressure was set at 1 atmosphere and temperature at 300 K. Like in NVT, hydrogen bonds constraints and LINCS algorithms were used. A leap-frog integrator with 2 fs time step was used. Production run simulations were run using NPT ensemble with hydrogen bonds constraints for the total of 20 ns each. Aside from the specific parameters indicated above, default settings were used otherwise.

## Results

### Protein structure assessment

**Fig. 1:**
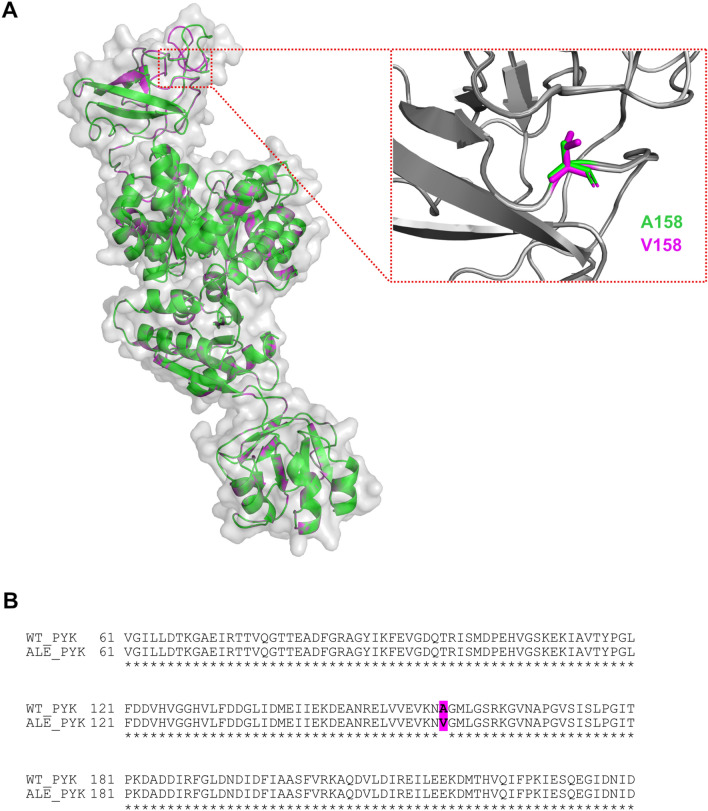
Protein model of pyruvate kinase. (**A**) Structural comparison between WT and ALE modelled protein structures and a close-up view on amino acid position 158, where substitution took place. (**B**) Part of the sequence alignment between WT and ALE PYKs, where position 158 is highlighted in magenta color.RMSD of the alpha-carbon (Ca) atoms as a function of time of WT (black) and ALE (red) PYKs at different temperatures

PYK of *E. faecium* is an oligomer, consisting of four identical subunits, and we used a single chain (594AA) for the analysis. We confirmed that ALE method introduced a mutation in the sequence at position 158, substituting Ala with Val (Fig. 1A and B). We utilized SWISS-MODEL for homology model construction and used PYK from *B. stearothermophilus* (PDB code: 2E28) as a template for both WT and ALE proteins (Fig. 1A). Upon construction of our models we used ProSA-Web server to assess the quality of the proteins (Wiederstein and Sippl 2007). Z-scores for WT and ALE proteins were identical and equal to − 11.21, which is an adequate score falling within the range of scores of other X-ray and NMR structures from databases (Wiederstein and Sippl 2007). Root-mean-square deviation (RMSD) score between two structures calculated using TM-align and was equal to 0.67 (Zhang 2005).

### Molecular dynamics simulations

The simulated heat treatment resulted in ALE strain to demonstrate better stability compared to that of the WT strain. Production MD simulations were performed at 300 K (27 °C), 340 K (67 °C), 350 K (77 °C) and 400 K (127 °C) for both WT and ALE pyruvate kinases for the duration of 20 ns per simulation. RMSD and RMSF data were plotted using complete trajectories to assess the stabilities of the proteins at different temperatures.

### Analysis of RMSD

**Fig. 2:**
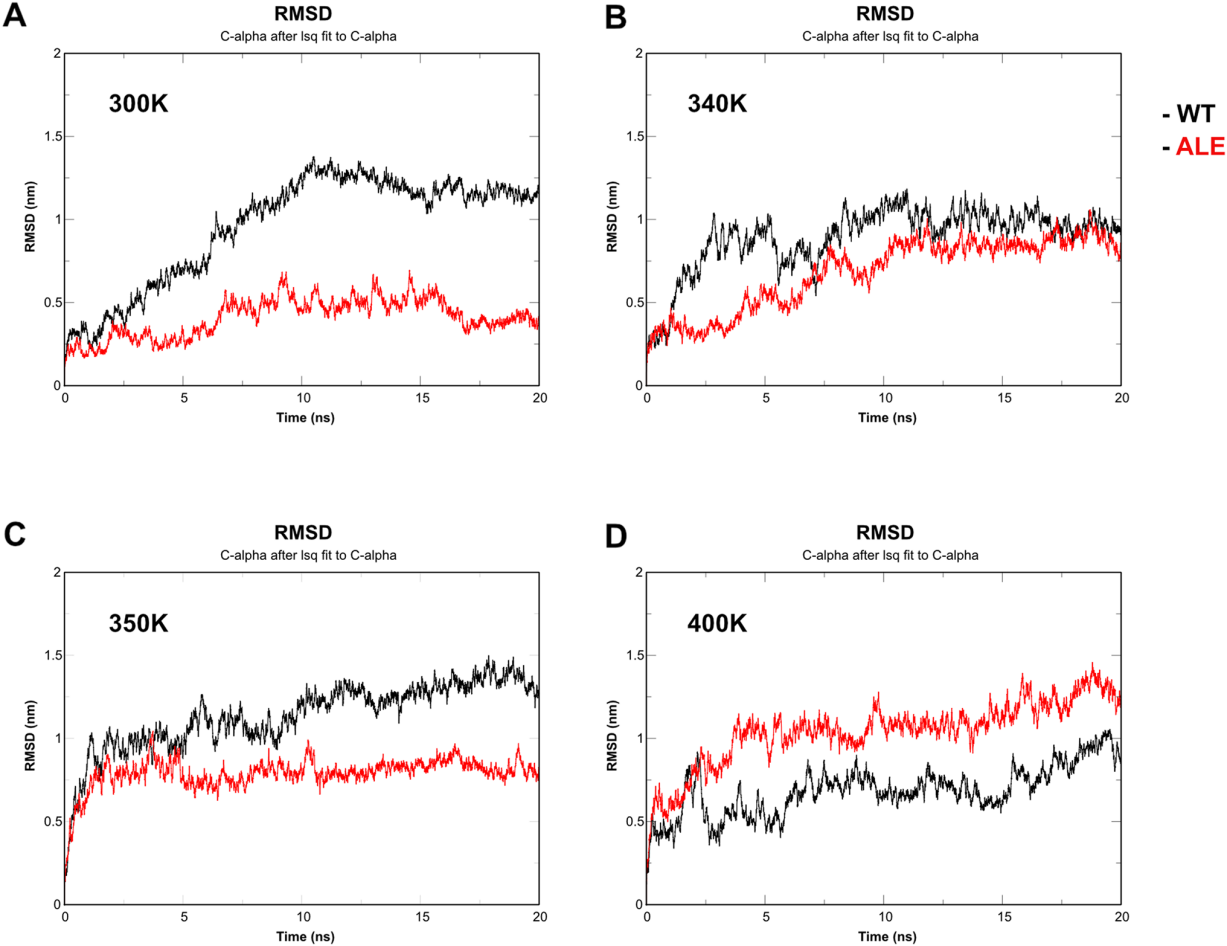
RMSD of the alpha-carbon (Ca) atoms as a function of time of WT (black) and ALE (red) PYKs at different temperatures. (**A**) 300K, (**B**) 340K, (**C**) 350K, (**D**) 400K

After performing MD simulations at 300 K it can be seen that RMSD values (Fig. 2A) showed a rapid increase to ~ 1.35 nm from 0 to 10 ns and stayed within a range of 1–1.3 nm for the rest of the simulation. ALE protein reached RMSD (Fig. 2A) value of 0.5 nm within first 6 ns and remained within a diapason between 0.3 and 0.6 nm for the remaining simulation. The overall average RMSD was 1.13 ± 0.2 nm. ALE PYK demonstrated less fluctuations, hence more stability at 350 K with average RMSD value 0.79 ± 0.09 nm (Fig. 2C). RMS deviations at 350 K were much higher for WT strain. For the first 10 ns, deviations were 1 ± 0.2 nm and then 1.2 ± 0.2 until the end of the simulation (Fig. 2C). ALE PYK exhibited at average RMSD of 1.04 ± 0.2 nm, while WT PYK had values of 0.69 ± 0.15 nm (Fig. 2D).

### Analysis of RMSF

**Fig. 3:**
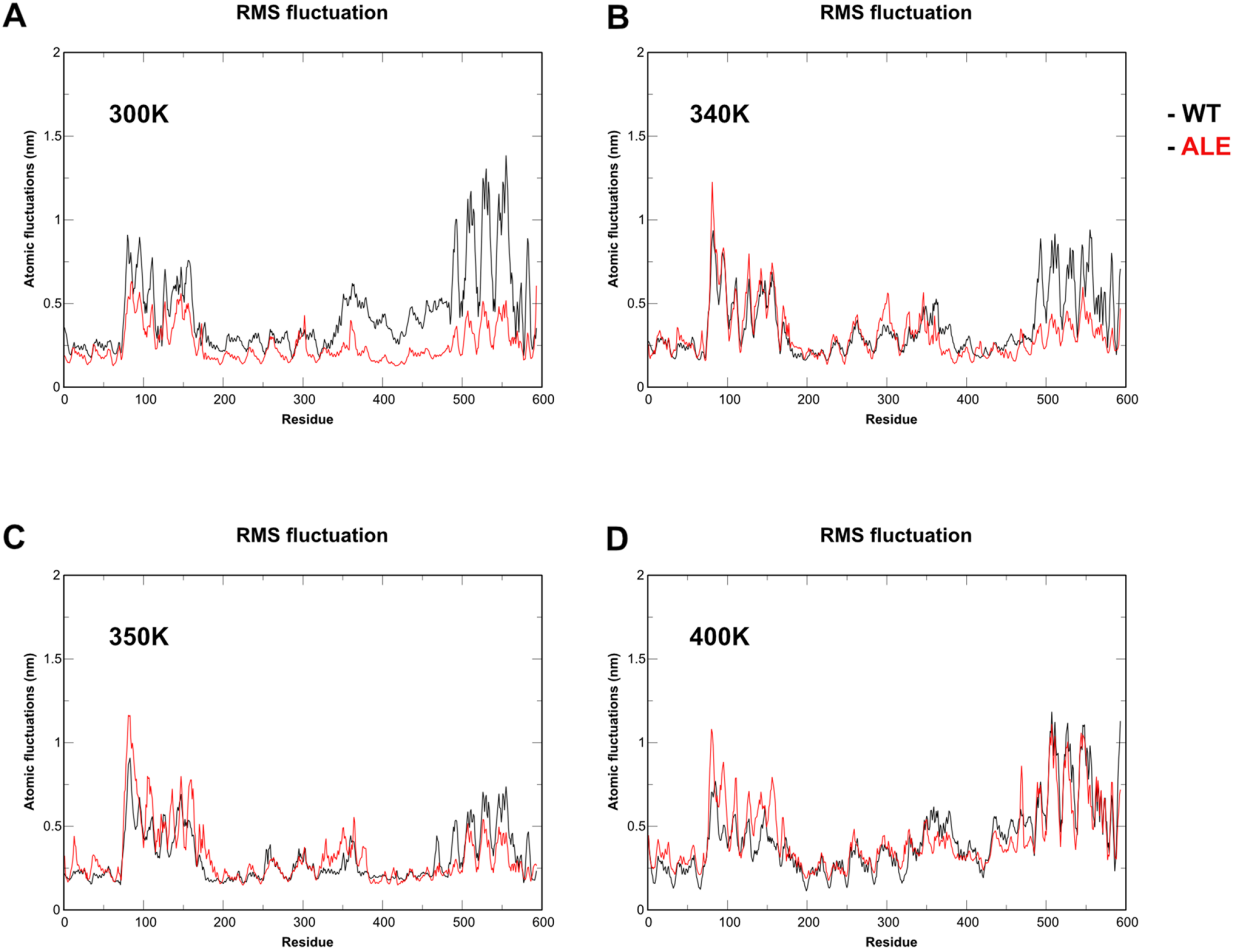
RMSF of the alpha-carbon (Ca) atoms from WT (black) and ALE (red) PYKs obtained at different temperatures. (**A**) 300K, (**B**) 340K, (**C**) 350K, (**D**) 400K

To determine regions responsible for deviations in RMSD plots, we prepared the root mean square fluctuation (RMSF) graphs of the Ca estimated at various temperatures (Table 1). Each individual graph represents the RMSF of WT and ALE PYKs (Fig. 3) at different temperatures. In all plots three distinct peaks can be observed: residues 70–180, 250–370 and 460–600. The first peak was the highest at 340 K (~ 1.25 nm) and belonged to ALE protein, while WT was around 0.9 nm. At temperatures other than 300 K (~ 0.9 nm) the peak was in range of ~ 0.7–1.1 nm and was primarily seen in ALE PYK. The highest peak was observed at 300 K (~ 1.4 nm) and was generated by WT PYK, while ALE protein showed deviations at only 0.5 nm. At temperatures of 340 and 350 K WT PYK showed higher fluctuations than ALE PYK, at around 0.9 nm and 0.6 nm correspondingly. ALE was at the level of 0.5 nm in both cases.

**Table 1:** RMSD and RMSF measured under different temperatures

Temperature	300 K		340 K		350 K		400 K	
	Average	Std. Dev.	Average	Std. Dev.	Average	Std. Dev.	Average	Std. Dev.
**RMSD**								
WT	0.96	0.33	0.89	0.19	1.14	0.2	0.69	0.15
ALE	0.4	0.11	0.68	0.21	0.79	0.09	1.04	0.2
**RMSF**								
WT	0.44	0.24	0.36	0.18	0.3	0.15	0.42	0.21
ALE	0.25	0.1	0.32	0.15	0.32	0.17	0.44	0.19

## Discussion

ALE technique is used extensively to develop enhanced strains of existing bacteria under specifically defined conditions. A significant advantage is offered in having bacteria undergo ALE method due to their relatively low nutrient requirements, simple cultivation and fast growth (Dragosits and Mattanovich 2013; Min et al. 2020). Previously Min et al. developed an ALE enhanced strain of *E. faecium* entitled BIOPOP-3 and discovered that identified genetic markers can be candidate markers closely associated with improved heat resistance of the new strain (Min et al. 2020). Five variants were identified in the following proteins: PYK, DNA/RNA helicases, exonuclease SbcC, KtrCD potassium uptake system and ribulosami ne/erythrulosamine 3-kinase. It is possible that one or several of the proteins mentioned above contributed to the newly acquired features of the ALE strain. In this work we focused on studying PYK and compared both WT and ALE strains to determine whether there is a significant difference in properties of these two kinases.

ALE protein demonstrated much higher stability than the WT at relatively low temperature (300 K), which can imply that the ALE strain developed by Min et al. improved not only the temperature resistance of bacteria but also the overall stability of the protein (Min et al. 2020). WT and ALE PYKs demonstrated similar RMSD values when tested at 340 K (Fig. 2B), achieving convergence at around 12 ns time point. After that the difference between the values was less than 0.5 nm for the rest of the simulation, implying that the ALE protein is more marginally stable than the WT protein at the given temperature. RMS deviations of WT at 350 K (Fig. 2C) were much higher than that at 340 K, implying lower stability of the protein. Based on the previous work ALE enhanced *E. faecium* demonstrated the most significant difference in survival rate at 75 °C, which is confirmed by our analysis (Min et al. 2020). The final simulation performed at an extreme temperature of 400 K was to detect, if present, unusual patterns of the proteins. WT protein was more stable than the ALE one, although, these data are arguable due to the fact that the original strains were approaching 0 survival rate at temperatures > 81 °C (Min et al. 2020). Therefore, we theorize that if 400 K temperature is applied to in vitro analysis neither WT nor ALE bacteria would survive the procedure. We believe that ALE demonstrated less deviations of its Ca at the widest range of temperatures (300 – 350 K), which indicates its contribution to the acquired thermostability of *E. faecium*.

The second peak was the highest at 300 K (~ 0.6 nm) and belonged to WT protein, while it was on the baseline level for ALE protein (Fig. 2A). Other temperatures showed, that the fluctuations were below 0.52 nm. The lowest flexibility of the protein was observed at 350 K and was distributed across the entire range of residues. The peaks were nearly identical for both WT and ALE PYKs at 400 K (~ 1 nm). Overall, region from 70 to 180 residues demonstrated the highest level of flexibility at 340 K (~ 1.2 nm) indicating that it is the most flexible at the given temperature. For the region 250–370 residues, the highest peaks were observed at 300 K (~ 0.6 nm), while they remained stable at the other temperatures (~ 0.5 nm). The highest peaks of the final Sects. (460–600 residues) were observed at 300 K (~ 1.4 nm).

To our knowledge this is the first work dedicated to analysis of PYK of *E. faecium* including homology modelling and MD simulation analysis. We performed a total of 20 ns MD simulations at each temperature point, and our results demonstrate that PYK from ALE enhanced *E. faecium* demonstrates better stability at 340 and 350 K. RMSF plots show that both proteins behave similarly and demonstrate a fair degree of flexibility at different temperatures. Although a better thermostability of PYK from ALE enhanced strain of *E. faecium* was proposed in this work, we believe that further analyses on all proposed proteins are required to draw a reasonable conclusion. Nevertheless, this work will prove beneficial in the *in silico* analysis of *E. faecium* and other commercially used bacteria.
